# Upper‐airway collapsibility and compensatory responses under moderate sedation with ketamine, dexmedetomidine, and propofol in healthy volunteers

**DOI:** 10.14814/phy2.14439

**Published:** 2020-05-22

**Authors:** Gaku Mishima, Takuro Sanuki, Shuntaro Sato, Masato Kobayashi, Shinji Kurata, Takao Ayuse

**Affiliations:** ^1^ Division of Clinical Physiology Department of Translational Medical Sciences Nagasaki University Graduate School of Biomedical Sciences Nagasaki Japan; ^2^ Department of Dental Anesthesiology Nagasaki University Hospital Nagasaki Japan; ^3^ Clinical Research Center Nagasaki University Hospital Nagasaki Japan

## Abstract

**Background:**

Ketamine is a potent sedative drug that helps to maintain upper‐airway patency, due to its higher upper‐airway dilator muscular activity and higher level of duty cycle, as seen in rats. However, no clinical trials have tested passive upper‐airway collapsibility and changes in the inspiratory duty cycle against partial upper‐airway obstruction in humans. The present study evaluated both the passive mechanical upper‐airway collapsibility and compensatory response against acute partial upper‐airway obstruction using three different sedative drugs in a crossover trial.

**Methods:**

Eight male volunteers entered this nonblinded, randomized crossover study. Upper‐airway collapsibility (passive critical closing pressure) and inspiratory duty cycle were measured under moderate sedation with ketamine, propofol, and dexmedetomidine. Propofol, dexmedetomidine, and ketamine anesthesia were induced to obtain adequate, same‐level sedation, with a BIS value of 50–70 and the OAA/S score of 2–3 and RASS score of −3.

**Results:**

The median passive critical closing pressure of 0.08 [−5.51 to 1.20] cm H_2_O was not significantly different compared to that of propofol sedation (−0.32 [−1.41 to −0.19] cm H_2_O) and of dexmedetomidine sedation (−0.28 [−0.95 to −0.03] cm H_2_O) (*p* = .045). The median passive *R*_US_ for ketamine 54.35 [32.00 to 117.50] cm H_2_O/L/s was significantly higher than that for propofol 5.50 [2.475 to 19.60] cm H_2_O/L/s; (mean difference, 27.50; 95% CI 9.17 to 45.83) (*p* = .009) and for dexmedetomidine 19.25 [4.125 to 22.05] cm H_2_O/L/s; (mean difference, 22.88; 95% CI 4.67 to 41.09) (*p* = .021). The inspiratory duty cycle increased significantly as the inspiratory airflow decreased in passive conditions for each sedative drug, but behavior differed among the three sedative drugs.

**Conclusion:**

Our findings demonstrate that ketamine sedation may have an advantage of both maintained passive upper‐airway collapsibility and a compensatory respiratory response, due to both increase in neuromuscular activity and the increased duty cycle, to acute partial upper‐airway obstruction.

## Graphical abstract

ketamine sedation may have an advantage of both maintained passive upper‐airway collapsibility and a compensatory respiratory response, due to the increased duty cycle, to acute partial upper‐airway obstruction.

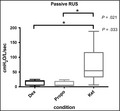

## 1 INTRODUCTION

Upper airway obstruction during sedation can result from changes in either the passive structural properties of the pharynx or from disturbances in neuromuscular control (Ayuse, 2010, 2016; Ayuse et al., 2009; Hoshino et al., 2009; Kobayashi et al., 2011), similar to the mechanism in sleep (Patil et al., 2007; Schneider et al., 2009). Methods for quantifying the contribution of anatomic alterations have been established in both sleeping and anesthetized subjects.(Eastwood, Platt, Shepherd, Maddison, & Hillman, 2005; Hoshino et al., 2009; Isono, Remmers, et al., 1997; Patil et al., 2007) When the upper‐airway first becomes obstructed, the upper‐airway musculature remains in a relatively hypotonic or *passive state* Patil et al., 2007; Schwartz et al., 1998) Initially, mechanoreceptor activity of the airway pressure receptors and pulmonary stretch receptors can produce immediate alterations in respiratory timing in experimental animals and sleeping humans. It has been suggested that a prolongation of the inspiratory duty cycle (IDC) can help stabilize ventilation during periods of upper‐airway obstruction (Hoshino et al., (2009); Schneider et al., 2009; Tagaito, Schneider, O'Donnell, Smith, & Schwartz, 2002), as a compensatory response against partial upper‐airway obstruction. Thereafter, upper‐airway obstruction can elicit compensatory neural responses that can mitigate the initial obstruction during spontaneous breathing in sleeping and in anesthetized subjects. With persistent upper‐airway obstruction, disturbances in gas exchange ensue, leading to increases in upper‐airway neuromuscular activity, improvements in airway patency, and greater ventilatory stability (*active state*) (McGinley et al., 2008; Patil et al., 2007; Schwartz et al., 1993; Seelagy et al., 1994). When compensatory mechanisms are inadequate to stabilize ventilation, upper‐airway obstruction often terminates in an arousal from sleep, with prompt restoration of upper‐airway patency (Younes, 2004). Arousals can therefore interfere with the assessment of compensatory upper‐airway and respiratory timing responses. We have previously reported that passive measurements of upper‐airway collapsibility in sedated subjects were similar to those in non‐REM sleep (Ayuse et al., 2004; Hoshino et al., 2009; Inazawa et al., 2005; Kobayashi et al., 2011). Active compensatory neuromuscular responses to partial upper‐airway obstruction have also been characterized in anesthetized subjects (Hoshino et al., 2009).

The protocols for sedation using propofol, dexmedetomidine, and ketamine are well established according to age, the magnitude of surgical invasion, and the site of the procedure, for maintaining safety in different clinical contexts. However, each of these drugs have different characteristics in terms of maintaining upper‐airway patency during sedation. Previously, Eikermann et al. (2012) strongly suggested that ketamine anesthesia can stabilize breathing by increasing the genioglossus activity, duty cycle, and respiratory rate, as compared with sleep and propofol‐induced unconsciousness, in a rat experiment. Furthermore, a recent study by Lodenius et al. (2019) indicated that dexmedetomidine and propofol exhibit similar degrees of pharyngeal collapsibility and reductions in ventilatory drive. They concluded that dexmedetomidine sedation does not inherently protect against upper‐airway collapse. However, no previous study had tested both the passive structural properties of the pharynx and neuromuscular compensatory control during sedation with different sedative drugs.

We hypothesized that at comparable levels of moderate sedation as assessed clinically, upper airway collapsibility is less with ketamine than that with propofol or dexmedetomidine. To address this hypothesis, we examined passive upper‐airway properties and timing responses to acute and sustained periods of airflow obstruction under ketamine, propofol, and dexmedetomidine sedation.

## 2 METHODS

### 2.1 Subjects

**Figure 1:**
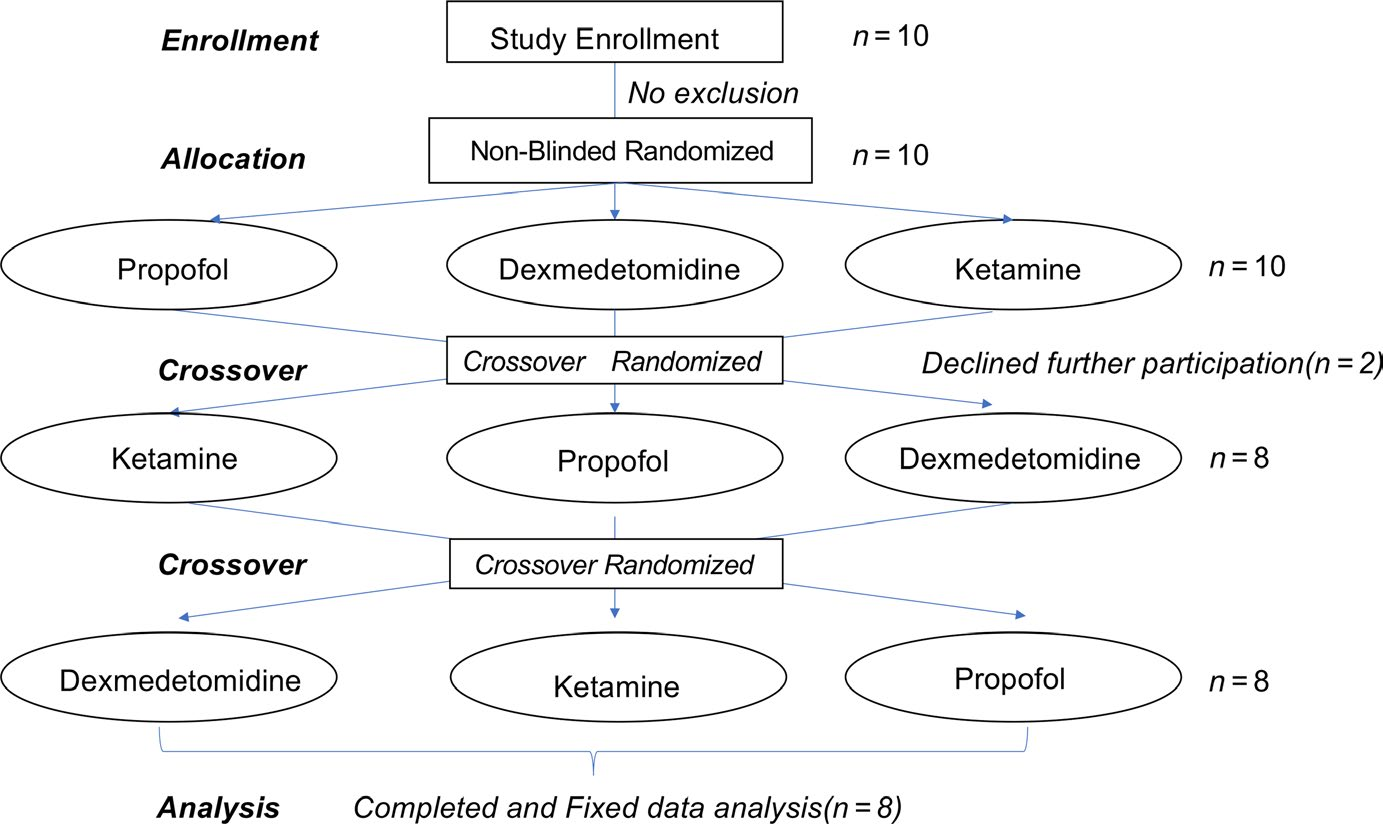
Standard flow diagram of the nonblinded, randomized crossover trial, showing the experimental enrollment criteria and number of individuals in the final analysis

All male participants with an American Society of Anesthesiologists physical status score of I or II were enrolled in this nonblinded randomized, crossover study (Figure 1). Because we have observed that there is a major influence of hormonal status in female subjects on upper airway patency, we have only recruited male subjects to eliminate these effects during sedation (Hoshino et al., 2011). Subjects were excluded if they were overweight or obese (body mass index more than 25 kg/m^2^), had a history of frequent or excessive snoring according to their bed‐partner, had abnormal sleep patterns assessed by Epworth Sleepiness score, or reported excessive daytime sleepiness (Epworth Sleepiness score > 10), or assessment of predisposition to sleep apnea by STOP‐BANG, had significant medical disease (cardiopulmonary pathology) or other clinical history (allergy to anesthetic), have a history of dysphagia, or reported tobacco use, or chronic alcohol or drug use. In addition, subjects were excluded if they had an anatomical deformation in the upper airway (e.g., deviated septum, retrognathia).

Upper‐airway pressure‐flow relationships were assessed under the awake condition and under moderate sedation with ketamine, dexmedetomidine, and propofol in eight male subjects (median age [range], 29.0 [24.5–34.75] yr; body weight, 64.5 [60.25–70.00] kg; height, 168.0 [165.3–172.3] cm; body mass index, 22.05 [21.17–24.15] kg/m^2^.

The protocol was approved by the Human Investigation Committee of the Nagasaki University School of Dentistry (No.0506–9) and registered with UMIN clinical trials registry (UMIN000038127). The enrollment of subjects was performed from September 2019 to January 2020 at the research facilities at Nagasaki University and written informed consent was obtained from all subjects.

### 2.2 Experimental procedures and monitoring

#### 2.2.1 Sedation level

All subjects underwent routine hemodynamic monitoring (systolic and diastolic blood pressure, as well as pulse rate). EEG signals were processed by the BIS monitor (Aspect Medical Systems, Inc., Natick, MA, USA) in order to determine the depth of anesthesia. The genioglossus electromyogram (EMG_GG_) was measured by surface electrode placed on the chin. Oxygen saturation was measured by pulse oximetry (SpO_2_). We required the subject's BIS value to be between 50 and 70 (Observer's Assessment of Alertness/Sedation (OAA/S) score 2–3: somnolence, response to tactile stimulation, or slow response to a loud voice) and that the subject scores a −3 on the Richmond Agitation‐Sedation Scale (RASS) in order to obtain an adequate and comparable moderate sedation level with each of propofol and dexmedetomidine. Because BIS is not a valid parameter for monitoring ketamine sedation, we used only the OAA/S score and the RASS scale to obtain a comparable moderate sedation level. At the conclusion of the measurements, all subjects remained in the supine position until they spontaneously emerged from anesthesia. Additionally, we will try to estimate the drug plasma concentration at stable sedative level 30 min after induction based on drug dosage and body weight, using the algorithm of pharmacokinetics of dexmedetomidine and ketamine using AnestAssist ^TM^ (Palma Healthcare Systems, USA).

#### 2.2.2 Respiratory measurements

Airflow (*V* ^¯^) was measured using a pneumotachometer (model 3,830, Hans Rudolph, Inc., Kansas City, MO, USA) and nasal pressure (*P*_N_) was measured using a differential pressure transducer (model 1100, Hans Rudolph, Inc.). The outflow from the valve attached to the nasal mask was then connected in series to the pneumotachometer and nasal mask, as described in a previous study (Kobayashi et al., 2011). Air leaks from the mouth were prevented by applying surgical tape across the lips. The changes in *P*_N_ could be made by utilizing a pressure generator (MAP/ResMed, Martinsried, Germany) operating over a−15 to +15 cm H_2_O range connected to the nasal mask. All measurements were displayed and recorded simultaneously on a computer using a data acquisition device (either Embla S7000 or A‐10, Medcare, Beverly Hills, CA, USA).

### 2.3 Experimental protocols

#### 2.3.1 Propofol sedation

No premedication was given. Propofol anesthesia was induced with intravenous propofol (Diprivan; Astra Zeneca, Nether Alderley, UK), administered via a Diprifusor (Astra Zeneca) target‐controlled infusion system (Terumo TCI pump TE371, Tokyo, Japan). The system calculated the effect site concentration on the basis of a three‐compartment pharmacokinetic algorithm (Coetzee, Glen, Wium, & Boshoff, 1995; Eastwood et al., 2005; Marsh, White, Morton, & Kenny, 1991). The propofol target blood concentration was increased and kept constant between 1.5 and 2.0 μg/ml to obtain an adequate level of anesthesia.

#### 2.3.2 Dexmedetomidine sedation

Dexmedetomidine was introduced at an initial load of 6 μg kg^‐1^ h^‐1^ for the first 10 min, and after 10 min, a dose of 0.2–0.7 μg kg^‐1^ h^‐1^ was used to maintain a constant sedation level.

#### 2.3.3 Ketamine sedation

Ketamine was introduced at an initial dose of 0.5–1.0 mg/kg, maintained at a rate of 0.05 mg kg^−1^ min^−1^, and supplemented with 0.5 mg/kg as needed to maintain a constant sedation level.

### 2.4 Respiratory parameters

Respiratory parameters were obtained under the baseline awake condition and under the sedated condition with each sedative drug. The respiratory parameters evaluated were as follows: respiratory rate (*f_R_*), minute ventilation (*V* ^¯^_E_), inspiratory duty cycle (IDC; IDC = *T*_I_/ *T*_TOT_), where *T*_I_ is the duration of inspiration and *T*_TOTAL_ is the duration of the inspiration and expiration, maximum inspiratory airflow (*V*_I_ max) during airflow‐limited breathing, and mean inspiratory airflow (*V*_I_ = *V*_T_/ *T*_I_), where *V*_T_ is the tidal volume. Dead space volume (*V*_DS_) was calculated using the formula, 24 × height^2^ (inches)/703 ml. Alveolar ventilation (*V* ^¯^_ALV_) was calculated by subtracting dead space ventilation, which is the product of *V*_DS_ and *f_R_*, from minute ventilation, as shown in the following equation:

(1) $${\bar{V}}_{\text{ALV}}={\bar{V}}_{E}-\left(V_{\text{DS}}\times f_{R}\right)$$

### 2.5 Primary outcome: upper airway collapsibility and passive critical closing pressure (P_CRIT_) during moderate sedation with ketamine, propofol, and dexmedetomidine

**Figure 2:**
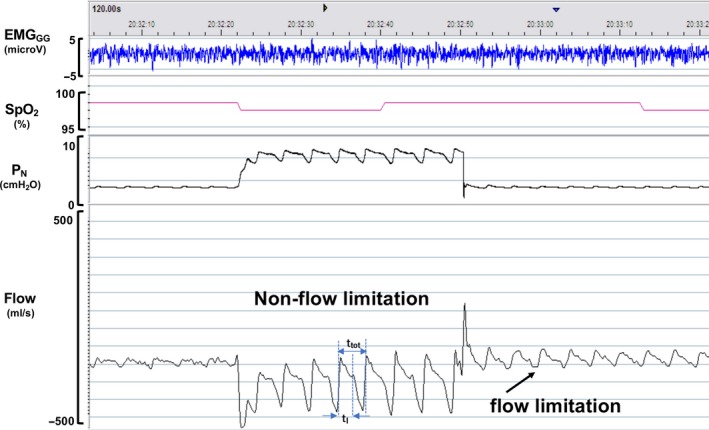
A schematic of the experimental protocol for producing upper airflow obstruction in the passive state. The polysomnographic recording in one subject is shown: surface genioglossus electromyogram (EM_GG_), oxyhemoglobin saturation (SpO_2_), nasal mask pressure (*P*_N_), and pneumotach airflow (Flow). *P*_N_ is abruptly reduced from an elevated holding pressure to a level that induced airflow limitation (plateau in airflow) within two or three steps, without causing an increase in EMG_GG_ activity and any reduction of SpO_2_ under sustained airflow limitation. This maneuver was repeated at least three times to generate passive pressure‐flow relationships. Note that, the drop in *P*_N_ from the holding pressure to lower pressure levels decreases the flow. The arrows indicate the occurrence of flow limitation as P_N_ decreases. T_tot_ (the duration of inspiration) and T_I_ (the duration of the inspiration and expiration) indicate each segment to calculate IDC in each breath

**Figure 3:**
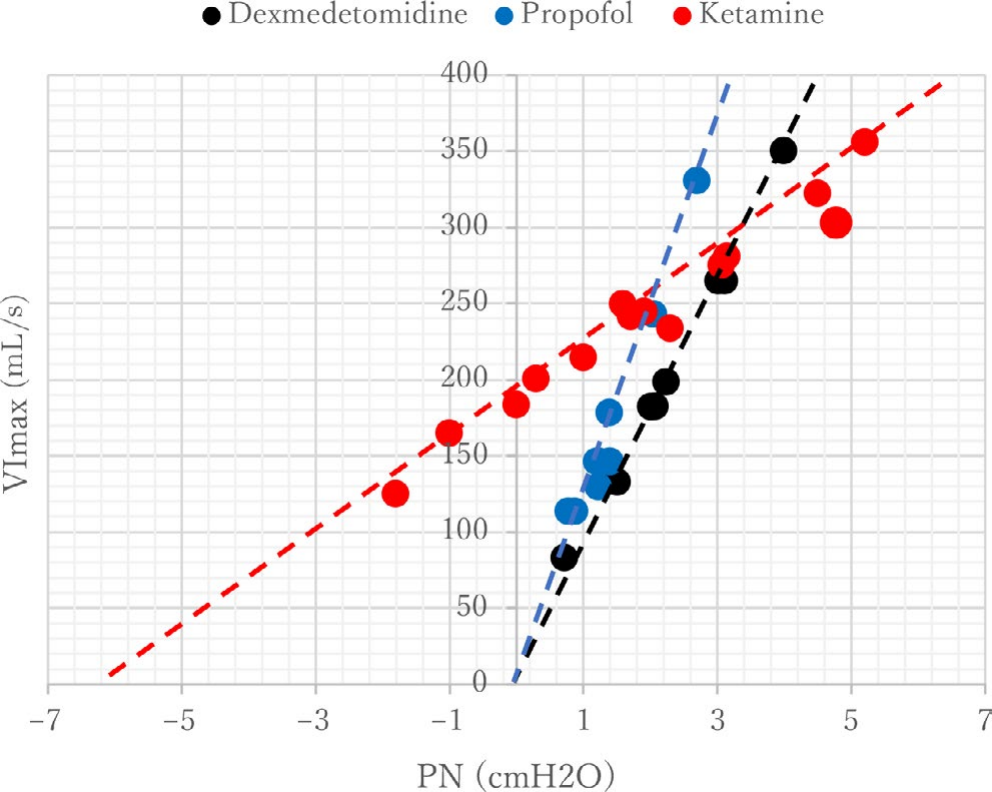
Representative trace of pressure‐flow relationships in one subject for each sedated condition: dexmedetomidine, propofol, and ketamine. The extrapolated estimated zero closing pressure was obtained by a linear regression curve between P_N_ and *V*_I_ max. The equation of the linear regression is *Y* = 117*x* + 1.2, *R* ^2^ = 0.95 for propofol, *Y* = 82*x* + 15, *R* ^2^ = 0.99 for dexmedetomidine, and *Y* = 29x + 186, *R* ^2^ = 0.97 for ketamine

Following the establishment of an adequate level of stable sedation, the subjects were initially allowed to breathe under atmospheric pressure. *P*_N_ was then gradually increased to a holding pressure where inspiratory airflow limitation was abolished (“passive state”), as previously described.(Boudewyns et al., 2000; Schwartz et al., 1998) To establish the passive *P*_CRIT_, *P*_N_ was rapidly lowered from the holding pressure to specific levels, for five successive breaths, before returning to the holding pressure (Figure 2a,b). Nasal pressure levels traversed a range of pressures, also including zero flow (airway occlusion). Passive pressure‐flow relationships were generated from at least two series of pressure drops over this range. The upper‐airway pressure‐flow relationship was analyzed to determine upper‐airway collapsibility. At each level of *P*_N_, breaths were evaluated for the presence of inspiratory airflow limitation, as previously described (Ayuse et al., 2004; Boudewyns et al., 2000; Hoshino et al., 2009, 2011; Kobayashi et al., 2011; Schwartz, Smith, Wise, Gold, & Permutt, 1988). Breaths that were associated with arousal evaluated with sudden change of increase in BIS value associated with change if raw EEG trace was excluded from analysis. Non‐flow‐limited breaths from the baseline condition at holding pressure were analyzed to determine the peak inspiratory airflow (*V*_I_ peak) at baseline. Maximal inspiratory flow (*V*_I_ max) and the corresponding *P*_N_ were obtained for each flow‐limited breath in the obstructed conditions. Least‐squares linear regression was used to generate the pressure‐flow relationship (Gold & Schwartz, 1996), which was fit by the following equation:

(2) $${\dot{V}}_{I}\max=\left(P_{N}-P_{\text{CRIT}}\right)/R_{\text{US}}$$

where *P*_CRIT_ is the estimated extrapolated zero critical closing pressure (*P*_N_ at zero flow) and *R*_US_ cm H_2_O/L/s is the resistSance of the portion of the tube upstream to the site of collapse (Figure 3).

### 2.6 Inspiratory duty cycle due to severity of upper‐airway obstruction

The IDC was obtained from non‐flow‐limited breaths at baseline and two levels of *V*_I_^¯^ obtained from breaths during passive conditions, as described in our previous study (Hoshino et al., 2009). The severity of upper‐airway obstruction was established based on the specific ranges of *V̄*_I_ above and below a clinically and physiologically relevant cutoff of approximately 150 ml/s. When *V̄*_I_ is below this cutoff, upper‐airway obstruction is known to lead to periodic obstructive hypopneas, whereas breathing patterns stabilize when *V*_İ_ exceeds this cutoff (Hoshino et al., 2009). Mild flow limitation was thereforeS defined by a *V*_I_ max of greater than 150 ml/s and severe flow limitation was approximated as a *V*_I_ max between 25 and 150 ml/s.

### 2.7 Sample size analysis

The sample size was calculated to determine what difference in *P*_CRIT_ was of clinical significance. We established the standard effect size by calculating individual differences from the raw data obtained from a previous study, which showed that head elevation with fixed‐jaw condition reduced *P*_CRIT_ to −7.2 ± 3.2 (mean ± *SD*) cm H_2_O, from a baseline of −2.8 ± 2.6 (mean ± *SD*) cm H_2_O in healthy volunteers (Kobayashi et al., 2011). Therefore, eight subjects were needed in each group to detect a difference in mean passive *P*_CRIT_ of 3.5–4.0 cm H_2_O with a type I error of 0.05 and a power of 0.9, based on a two‐tailed paired *t*‐test. Anticipating a drop‐out rate of 10%–15%, we enrolled 10 subjects in each group.

Subjects were nonblinded and were randomly scheduled to undergo sedation with propofol, dexmedetomidine, or ketamine by an independent researcher using an envelope method. A crossover design was used for all subjects, with at least a 1‐week interval between each sedative drug administration. The allocation sequence was generated by the statistician, and the research staff opened the sequentially numbered envelope containing the randomization assignment. The research assistant evaluated eligibility, obtained informed consent, and enrolled the participants in this study.

### 2.8 Statistical analysis

Continuous normal distribution data are presented as median [interquartile range] or mean ± *SD*. Categorical nonparametric data are presented as median [interquartile range] or numbers. Normal distribution of data was assessed using histograms and Q‐Q plots. To evaluate how obstruction level, drug type, and obstruction level × drug interaction affected IDC results, data were analyzed using a linear mixed model. We also set the drug type and obstruction level as fixed effects and individuals as a random effect. In post hoc testing, Tukey's multiple comparison test was applied to examine differences compared with drug type by obstruction level, and obstruction level by drug level. The passive *P*_CRIT_, *R*_US_ for each drug and the value of *V*_T_, *f_R_*, *V̄*_E_, *V̄*_ALV_, and SpO_2_ between the awake and sedative condition for each drug were analyzed by Student's paired *t*‐test. Statistical analysis was performed with R (version 3.2.4, R Foundation for Statistical Computing, Vienna, Austria). A *p*‐value of less than .05 was considered to be statistically significant.

## 3 RESULTS

Complete data sets were obtained from eight subjects and there was no missing data.

### 3.1 Effect of sedation on breathing parameters under THREE sedated conditions

For propofol sedation, the mean target blood concentration of propofol was 1.54 ± 0.23 μg/ml propofol, which produced adequate anesthesia and a BIS score of 66.3 ± 2.4. There was no significant difference in the BIS score between propofol and dexmedetomidine (64.3 ± 9.3, *p* = .33). An OAA/S score of 2–3 was confirmed for all three sedative drugs: average 2.3 ± 0.5 for propofol, 2.5 ± 0.5 for dexmedetomidine, and 2.5 ± 0.5 for ketamine. An RASS scale of −3 was confirmed for all three sedative drugs, indicating that comparable levels of moderate sedation were achieved with the value of average −3.0 ± 0.5 for propofol, −2.9 ± 0.4 for dexmedetomidine, and −2.8 ± 0.5 for ketamine. We obtained estimated plasma concentration of approximately 575.38 ± 48.89 ng/ml in ketamine sedation and approximately 0.61 ± 0.11 ng/ml in dexmedetomidine. We used TCI for propofol of 1.5 ~ 2.0 μg/ml.

Respiratory parameters, such as *V* ^˙^_I_ max, *f*_R_, *V*T, *V̄*_E_, and *V̄*_ALV_, are represented for the baseline awake condition and each sedated condition in Table 1. During stable sedated conditions with each of the sedative drugs, *f*_R_ was significantly higher than during the awake condition. *V*_T_ was significantly decreased under all sedated conditions than under the awake condition. There was no significant difference in *V̄*_E_ and *V̄*_ALV_ under sedation compared to under quiet wakefulness.

**TABLE 1:** Respiratory parameters for baseline awake condition and the sedative condition, with each sedative agent.

	Propofol		Dexmedetomidine		Ketamine	
	Awake	Sedation	Awake	Sedation	Awake	Sedation
*V_T_*	359 ± 51	268 ± 108*	357 ± 83	271 ± 50*	360 ± 38.5	283 ± 76*
*f*_R_	12.5 ± 4.7	18.4 ± 3.4*	11.1 ± 4.4	18.0 ± 2.3*	12.8 ± 4.3	14.0 ± 4.3
${\bar{V}}_{E}$	4518 ± 1644	4747 ± 1145	3833 ± 1190	4804 ± 658	4602 ± 1730	3606 ± 1314
${\bar{V}}_{\mathrm{ALV}}$	2608 ± 910	1983 ± 1263	2137 ± 792	2081 ± 913	2662 ± 1111	1928 ± 1027
SpO_2_	98.8 ± 1.3	97.0 ± 1.1	98.6 ± 1.1	96.4 ± 2.0	98.3 ± 1.3	98.3 ± 1.9
BIS		66.3 ± 2.4		64.3 ± 9.3		
OAA/S		2.3 ± 0.5		2.5 ± 0.5		2.5 ± 0.5

The asterisk (*) indicates significant differences from the awake condition; *p* < .05. There is a significant difference in the respiratory rate (*f*_R_) under sedation with propofol and dexmedetomidine, but not with ketamine. There is a significant difference in the tidal volume (*V*_T_) among the three sedative conditions. There is no significant difference in alveolar ventilation ( ${\bar{V}}_{\mathrm{ALV}}$)among the sedative drugs.

### 3.2 Passive upper‐airway properties

**Figure 4:**
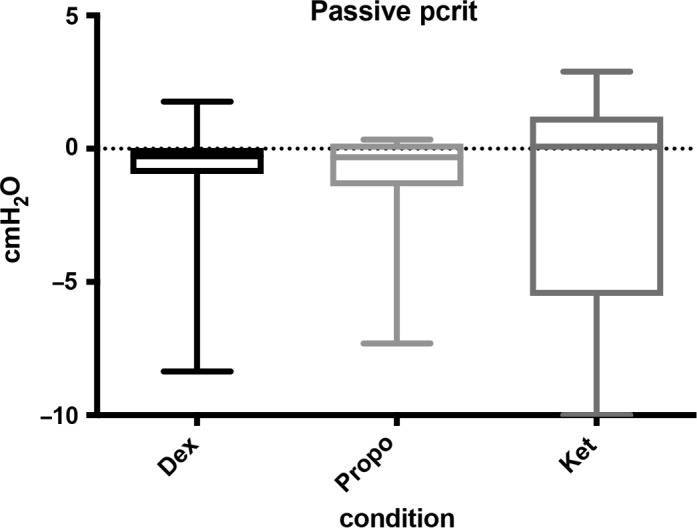
The median passive *P*_CRIT_ values for each sedation condition are shown in Figure 4. The median passive *P*_CRIT_ for ketamine [interquartile range], 0.08 [−5.51 to 1.20] cm H_2_O, was not significantly different compared to that of propofol −0.32 [−1.41 to −0.19] cm H_2_O (mean difference, −2.59; 95% CI −4.52 to −0.66) (*p* = .016) and dexmedetomidine −0.28 [−0.95 to −0.03] cm H_2_O (mean difference, −0.13; 95% CI −1.37 to 1.11) (*p* = .045). There was no significant difference in *P*_CRIT_ between propofol and dexmedetomidine (*p* = .812)

**Figure 5:**
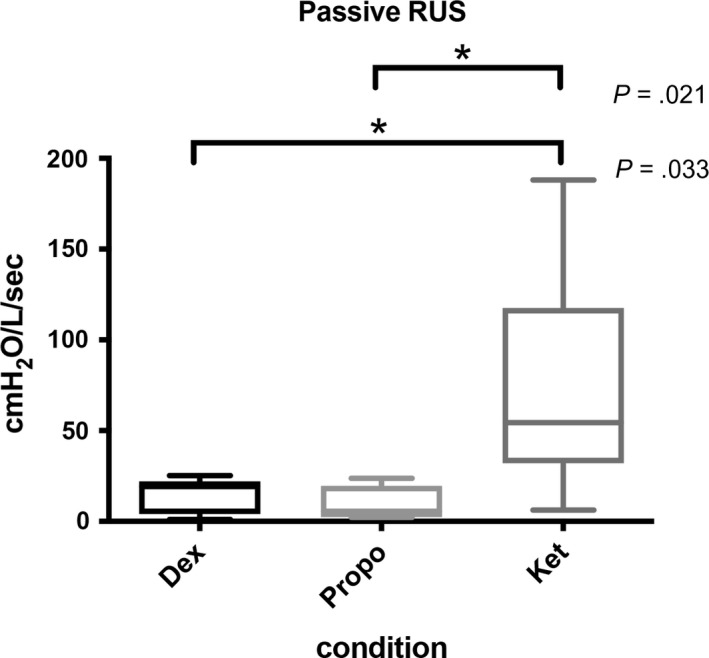
The median passive *R*_US_ for ketamine 54.35 [32.00 to 117.50] cm H_2_O/L/s was significantly higher than that for propofol 5.50 [2.475 to 19.60] cm H_2_O/L/s; (mean difference, 27.50; 95% CI 9.17 to 45.83) (*p* = .009) and for dexmedetomidine 19.25 [4.125 to 22.05] cm H_2_O/L/s; (mean difference, 22.88; 95% CI 4.67 to 41.09) (*p* = .021) (Figure 5). There was no significant difference in *R*_US_ between propofol and dexmedetomidine (*p* = .133)

The median passive *P*_CRIT_ values for each sedation condition are shown in Figure 4. The median passive *P*_CRIT_ for ketamine [interquartile range], 0.08 [−5.51 to 1.20] cm H_2_O, was not significantly different compared to that of propofol −0.32 [−1.41 to −0.19] cm H_2_O (mean difference, −2.59; 95% CI −4.52 to −0.66) (*p* = .016) and dexmedetomidine −0.28 [−0.95 to −0.03] cm H_2_O (mean difference, −0.13; 95% CI −1.37 to 1.11) (*p* = .045). There was no significant difference in *P*_CRIT_ between propofol and dexmedetomidine (*p* = .812). The median passive *R*_US_ for ketamine 54.35 [32.00 to 117.50] cm H_2_O/L/s was significantly higher than that for propofol 5.50 [2.475 to 19.60] cm H_2_O/L/s; (mean difference, 27.50; 95% CI 9.17 to 45.83) (*p* = .009) and for dexmedetomidine 19.25 [4.125 to 22.05] cm H_2_O/L/s; (mean difference, 22.88; 95% CI 4.67 to 41.09) (*p* = .021) (Figure 5). There was no significant difference in *R*_US_ between propofol and dexmedetomidine (*p* = .133).

### 3.3 Compensatory response to acute and sustained periods of airflow obstruction

**Figure 6:**
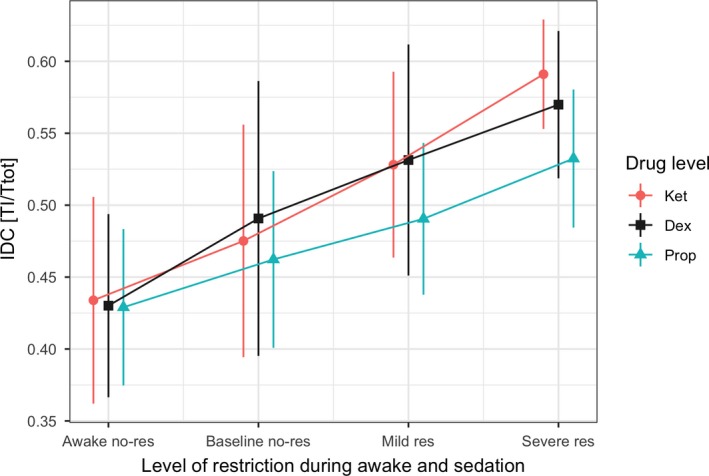
Compensatory response to acute and sustained periods of airflow restriction (awake no‐restriction, baseline no‐restriction, mild restriction, and severe restriction) was evaluated in terms of change in inspiratory duty cycle (IDC) under each sedative condition. The change in IDC at different levels of *V̄*_I_ under each sedative condition is shown. The IDC increased significantly, as *V*_I_ decreased in passive conditions for each sedative drug. There is a significant difference in the IDC response between propofol and ketamine (*p* = .025), and between propofol and dexmedetomidine (*p* = .013) at a mild restriction level. There is also a significant difference in the IDC response between propofol and ketamine (*p* < .001), and between propofol and dexmedetomidine (*p* = .025) at the severe restriction level. There is significant difference in IDC between awake and baseline no‐restriction, mild restriction, and severe restriction levels for all three sedation drugs (*p* < .001). There is no significant difference between baseline and mild restriction during propofol sedation (*p* = .196). awake no‐restriction (awake no‐res), baseline no‐restriction (baseline no‐res), mild restriction (mild res), and severe restriction (severe rest), propofol (Prop), dexmedetomidine (Dex), ketamine (Ket)

The compensatory response to acute and sustained periods of airflow obstruction (baseline non‐flow‐limited, mild obstruction, and severe obstruction) was evaluated in terms of changes in IDC for each sedated condition. Figure 6 shows the change in IDC for different levels of *V̄*_I_ under each sedated condition. The IDC increased significantly as *V*_I_ max decreased in passive conditions, for each sedative drug. In the comparison of drug type by obstruction level, there was a significant difference in IDC response between propofol and ketamine (*p* = .025), and propofol and dexmedetomidine (*p* = .013) at a mild obstruction level. There was a significant difference in the IDC response between propofol and ketamine (*p* < .001), and between propofol and dexmedetomidine (*p* = .025) at a severe obstruction level. When we compared the obstruction level by the drug level, there was a significant difference (*p* < .001) among obstruction levels for all three sedation conditions, except between baseline and mild obstruction during propofol sedation (*p* = .196).

## 4 DISCUSSION

The present study compared both passive mechanical upper‐airway collapsibility and compensatory responses to acute partial upper‐airway obstruction under three different sedative drugs. Ketamine sedation maintained upper‐airway patency with similar collapsibility, as assessed by passive *P*_CRIT_, compared to propofol and dexmedetomidine sedation. However, the mean passive *R*_US_ for ketamine was significantly higher than propofol and dexmedetomidine. The activated compensatory response to partial upper‐airway obstruction, as demonstrated by increased IDC, remained intact with ketamine. Upper‐airway obstruction induced immediate, progressive IDC increases with increased airflow obstruction (*V̄*_I_) severity, for all three sedative drugs. As confirmed by the existence of compensatory response to restore ventilation during non‐REM sleep (Patil et al., 2007), the compensatory ventilatory responses as well as timing parameters were partially preserved with ketamine, similarly or superior to propofol and dexmedetomidine sedation.

There are substantial increases in pharyngeal collapsibility (Patil et al., 2007) in spontaneously breathing patients during sedation and anesthesia (Drummond, 1996; Oshima, Masaki, & Toyooka, 1999). Although anesthesia and/or neuromuscular blockade can elevate upper‐airway collapsibility (Eastwood et al., 2005; Isono, Tanaka, Tagaito, Sho, & Nishino, 1997b), passive *P*_CRIT_ (−3.8 cm H_2_O) under ketamine sedation was comparable to that during non‐REM stage 2 sleep (−4.5 cm H_2_O) in healthy subjects (Kirkness et al., 2008; Patil et al., 2007). In contrast, the *P*_CRIT_ under propofol (−1.2 cm H_2_O) and dexmedetomidine sedation (−1.1 cm H_2_O) was similar to that under midazolam sedation (−1.9 cm H_2_O) (Ayuse et al., 2006), and was more negative than that under isoflurane anesthesia (1.2 cm H_2_O) (Eastwood, Szollosi, Platt, & Hillman, 2002), which produced notable decreases in pharyngeal neuromuscular tone and patency. The passive *P*_CRIT_ values of this study were consistent with the reported values for moderate sedation with dexmedetomidine (0.3 [−9.2 to 1.4] cm H_2_O) and propofol (−0.6 [−7.7 to 1.3] cm H_2_O) (Lodenius et al., 2019). Therefore, it is likely that hypotonic pharynx collapsibility during ketamine sedation is similar to that observed during natural non‐REM sleep. Passive upper‐airway patency may be moderately influenced under propofol and dexmedetomidine sedation.

Interestingly, R_US_ was significantly different under ketamine compared to propofol and dexmedetomidine sedation, indicating resistance of the airway region upstream to the site of collapse. We had not found a R_US_ change in our previous study, although *P*_CRIT_ differed significantly. This may indicate increased muSscle activity in the upper‐airway dilator muscle, as suggested by Eikermann et al. (2012) Upper‐airway dilator muscle (e.g., genioglossus) activation can mitigate airway obstruction (Fogel et al., 2001; Malhotra et al., 2000; Mezzanotte, Tangel, & White, 1992). Despite depression of dilator activity under propofol anesthesia, dilator responses to sustained upper‐airway obstruction were evident, and could account for increased upper‐airway patency (increased *V̇*_I_ max) and decreased *P*_CRIT_. These responses could also have facilitated *V*_I_ max maintenance under the mildly flow‐limited active condition, at levels comparable to baseline (non‐flow‐limited) *V*_I_ peak levels. Airflow became limited during sustained reductions in nasal pressure, because negative tracheal pressure swings increased as ventilatory drive increased. Decreased airflow caused by nasal pressure reduction (Schwartz et al., 1988) might reflect progressive decreases in intraluminal pressures (Rowley, Williams, Smith, & Schwartz, 1997). These findings imply that neuromuscular responses stabilized upper‐airway patency during sustained airflow obstruction. The time‐course of these compensatory responses is more consistent with chemoreceptor than with mechanoreceptor stimulation (Seelagy et al., 1994).

During periods of inspiratory airflow limitation, the IDC increased immediately and progressively as airflow obstruction severity increased during sedation, consistent with the time‐course of mechano‐ rather than chemoreflex responses (Chow, Xi, Smith, Saupe, & Dempsey, 1994; Dejours, 1962; Iber, Simon, Skatrud, Mahowald, & Dempsey, 1995; Kimoff, Sforza, Champagne, Ofiara, & Gendron, 2001; Leevers, Simon, Xi, & Dempsey, 1993; Manchanda et al., 1996). The increased IDC (*T*_I_/ *T*_TOT_) restores ventilation at any given level of upper‐airway obstruction, as described by the relationship: *V̄*_E_ = *V*_T_ / *T*_I_ × *T*_I_ / *T*_TOT_, where *V*_T_/*T*_I_ represents the mean inspiratory flow rate. The IDC initially increased with upper‐airway obstruction development and decreased as obstruction abated (as *V̇*I max rose), with the activation of pharyngeal neuromuscular responses (Schneider et al., 2003, 2009; Tagaito et al., 2002). Thus, distinct physiological mechanisms govern the IDC and upper‐airway responses to airway obstruction. The IDC response offers immediate relief from nocturnal hypoventilation during upper‐airway obstruction periods in sleep (Schneider et al., 2009), and is partially preserved during propofol, dexmedetomidine, and ketamine sedation. Interestingly, as the passive mechanical properties are similar under propofol and dexmedetomidine sedation, the IDC response to partial upper‐airway obstruction is also similar to the response under dexmedetomidine sedation, which has a higher baseline IDC value. Furthermore, the IDC response under ketamine sedation is more functional, as indicated by the steeper IDC response curve, than that with other sedative drugs. During sedation, ventilation can only be preserved by prolonging the IDC (Schneider et al., 2003; Younes, 2003), which maintains and stabilizes ventilation during periods of inspiratory flow limitation. We observed increases in *f*_R_ (Table 1) for a given IDC under sedated conditions, which would decrease *V*_T_, increase the dead space fraction, and decrease *V*_ALV_ accordingly. Although we did not observe any significant *V*_ALV_ reduction during sedation, compared to under the awake condition, *V*_ALV_ might change as the upper‐airway becomes obstructed under moderate to deep sedation. Thus, IDC and *f_R_* responses to a given level of upper‐airway obstruction may determine the degree of hypoventilation during sedation. This IDC response could be an advantage of dexmedetomidine and ketamine sedation, providing a well‐preserved compensatory response to acute partial upper‐airway obstruction.

There were several limitations in this study. For example, the results may be imprecise due to errors in outcome measurements, misdiagnosis, or misclassification of events. The potential influence of sources of bias may also substantially impact the interpretation of the trial.

First, we could not produce maximum activation of the respiratory response based on IDC measurement, because subjects’ exposure to hypoxemia was limited for ethical reasons. This may have attenuated the degree of activation during the stepwise decreases in nasal pressure, leading to underestimation of the full strength of the active response based on IDC changes.

Second, sedation levels, based on evaluation of the OAA/S score, RASS score, and BIS monitoring, with propofol, dexmedetomidine, and ketamine might differ slightly pharmacologically. However, the BIS value obtained in this study was consistent with that previously reported for propofol and dexmedetomidine (Lodenius et al., 2019). That study reported BIS at the time of critical pharyngeal pressure measurements as 57 ± 16 and 39 ± 12 during moderate infusion rates of dexmedetomidine and propofol, respectively. Therefore, we considered the sedation level obtained in this study (BIS: 50–70, OAA/S score 2–3, and RASS score −3) as a clinically relevant moderate sedation level. Third, this study did not reveal any severe adverse events associated with ketamine‐only administration that may counter the potential benefit of maintained airway patency including dysphoria and increased salivation/secretions; however, the patients were monitored for these adverse events for only a short period of time.

Fourth, the differences in upper airway mechanics could really be due to subtle differences between the drugs’ mode of anesthetic action, despite ostensible similarities in clinical sedation level. In this study, we found the maintained a compensatory response as an increase in the IDC and increase in neuromuscular activity as an increase in *R*_US_ in ketamine sedation. Although the combination of two fundamentally different neural compensatory mechanisms, that is instantaneous prolongation of the IDC and increase neuromuscular activity, might be the most important factor for both maintaining passive upper airway collapsibility and ventilation sedation, further study to test different level of compensatory mechanism depends on anesthetic depth.

Our findings have two major clinical implications.

First, in addition to the advantage of ketamine over propofol and dexmedetomidine in maintaining passive upper airway patency in a given clinical setting, ketamine may also be more suitable than propofol for sedation during invasive procedures due to its synergistic effect with local anesthesia. However, it has been suggested that ketamine should be used in combination with benzodiazepines in order to avoid the side effects of ketamine, such as nightmares; the effects of such combinations on upper‐airway patency should be further investigated. Drummond (1996) suggested that ketamine had beneficial effects on airway patency. Furthermore, ketamine is recommended in difficult airway situations where spontaneous respiration needs to be preserved in adults (Craven, 2007). In critically ill children, a lower incidence of airway obstruction was observed under ketamine than under propofol sedation (Vardi, Salem, Padeh, Paret, & Barzilay, 2002). Ketamine combines potent analgesic with hypnotic actions (Bourgoin et al., 2003). Further research is needed to test upper‐airway patency under ketamine sedation combined with other agents such as potent analgesics with hypnotic actions.

Second, the preservation of compensatory neuromuscular responses involved in respiratory control suggests that upper‐airway patency can be restored during sedation with all three sedative drugs, and particularly with ketamine sedation. Our previous study suggested that immediate IDC responses can prevent a precipitous decrease in ventilation during propofol anesthesia, whereas more sustained periods of upper‐airway obstruction in the absence of arousal are required for the restoration of upper‐airway patency (Hoshino et al., 2009).

Ketamine sedation may preserve upper‐airway patency, although the effect of ketamine on upper‐airway patency in humans is poorly understood at present. Eikermann et al. (2012) hypothesized that spontaneously breathing animals, when administered ketamine, maintain breathing by two mechanisms: augmentation of airway dilator muscle activity and increased IDC. The IDC determines ventilatory stability; an increase in the IDC can compensate for partial airway obstruction Schneider et al. (2009). Ketamine induces a dose‐dependent increase in the IDC. This mechanism can compensate for partial airway obstruction (Schneider et al., 2009). Of note, in a previous study, a dose‐dependent IDC increase was observed in animals breathing normally. Accordingly, we concluded that the increase in IDC was not a compensatory mechanism for partial upper‐airway obstruction, but rather represented a direct and beneficial drug effect on inspiratory time.

Although the threshold for arousal responses and surface genioglossus electromyogram (EMG_GG_) activation is significantly depressed with propofol, some compensatory mechanisms may still persist. Previously, Eastwood et al.(2005) reported that increasing depths of propofol anesthesia are associated with increased upper‐airway collapsibility. Furthermore, Lodenius et al.(2019) indicated that passive pharyngeal critical pressure may have similar effects during low or moderate sedation with dexmedetomidine and propofol. Although they concluded that dexmedetomidine sedation does not protect against upper airway collapse, there might be some advantage of maintaining a compensatory response to partial upper airway obstruction, based on our finding regarding IDC response. They concluded that dexmedetomidine sedation does not protect against upper‐airway collapse. We strongly support their suggestion that continuous respiratory monitoring, such as capnography and pulse oximetry, is advisable during any level of sedation, to detect early stages of upper‐airway obstruction. Furthermore, risk factors of positional change and background disease, such as obstructive sleep apnea syndrome, should be considered in future studies.

In conclusion, we found that passive upper airway patency with less collapsibility is maintained and compensatory neuromuscular responses to upper‐airway obstruction remained intact during ketamine sedation, as compared to a similar level of propofol or dexmedetomidine sedation. Moreover, distinct mechanisms underlie ventilation maintenance during upper‐airway obstruction, with specific latencies to responses in the IDC (immediate) and inspiratory airflow (delayed). Partitioning the upper‐airway properties into structural and neuromuscular components could facilitate establishing ways to evaluate and maintain upper‐airway patency and ventilation under sedation with different anesthetic agents, and ultimately prevent perioperative respiratory complications of anesthesia.

## CONFLICT OF INTEREST

GM., TS., SS., MK, SK., and TA. have no declaration of interest.

## AUTHOR CONTRIBUTIONS

GM, TS, SK, MK, and TA are responsible for conceiving and designing the trial, planning data analysis, drafting the manuscript, and approving the final manuscript. GM and TS are responsible for participating in data collection and are in charge of recruitment and treatment of patients. SS is responsible for planning data analysis and analyzing the data resulting from the trial. All authors will have access to the interim results as well as the capacity to discuss, revise, and approve the final manuscript.
